# Role of Albumin as a Nutritional and Prognostic Marker in Elective Intestinal Surgery

**DOI:** 10.1155/2020/7028216

**Published:** 2020-04-13

**Authors:** Christian Galata, Linda Busse, Emrullah Birgin, Christel Weiß, Julia Hardt, Christoph Reißfelder, Mirko Otto

**Affiliations:** ^1^Department of Surgery, Universitätsmedizin Mannheim, Medical Faculty Mannheim, Heidelberg University, Mannheim, Germany; ^2^Department of Medical Statistics and Biomathematics, Medical Faculty Mannheim, Heidelberg University, Mannheim, Germany

## Abstract

**Background:**

The aim of this study was to investigate albumin, nutritional status, and inflammation in the perioperative course of patients undergoing elective intestinal surgery.

**Methods:**

A retrospective analysis of patients with preoperative measurements of nutritional parameters who underwent intestinal surgery between April 2017 and August 2018 at our institution was performed. Preoperatively, the correlation of albumin levels with markers for inflammation and nutritional status was investigated. Postoperatively, albumin levels were assessed with regard to high-grade morbidity and inflammation.

**Results:**

A total of 105 patients were included. Preoperatively, albumin levels were correlated with both markers for nutritional status and inflammation, with phase angle (PA) (*p*=0.004) and C-reactive protein (CRP) (*p* < 0.001) as independent factors predicting the albumin levels in multivariable analysis. Postoperatively, the reduction in serum albumin (∆-albumin) on postoperative day (POD) 1/2 (*p*=0.025) and POD 4/5 (*p*=0.003) was significantly associated with Clavien–Dindo complications ≥grade III. A cut-off value of 27.3% for ∆-albumin on POD 1/2 predicted postoperative high-grade morbidity (sensitivity 75% and specificity 69%). The product of ∆-albumin and CRP on POD 4/5 identified patients with major complications more reliably than ∆-albumin or CRP alone (sensitivity 91% and specificity 72%).

**Conclusion:**

Preoperatively, albumin was a marker for nutritional status even if an inflammatory component was present. Postoperatively, ∆-albumin on POD 1/2 predicted high-grade morbidity. A new marker to identify patients with major complications on POD 4/5 is presented.

## 1. Introduction

Hypoalbuminemia is a powerful predictor of postoperative morbidity in patients undergoing gastrointestinal surgery for malignancies or inflammatory bowel diseases (IBD) [1, 2]. Low levels of albumin have long been observed in patients with malnutrition [3, 4]. Traditionally, albumin has been regarded as a nutritional marker, a fact that leads to the administration of enteral or parenteral nutritional therapy in patients with hypoalbuminemia in various situations in clinical practice, e.g., preoperatively [5–7]. However, the idea that albumin reflects nutritional status is controversial. While a number of studies have regarded albumin as a nutritional parameter [4, 5, 8–11], other authors have questioned this relation, especially in healthy or nonsurgical patients [12–15]. In most clinical cohorts, low albumin levels are the result of the combined effects of inflammation and inadequate caloric intake [16]. Although the importance of albumin as a prognostic marker for clinical outcomes in surgical patients is well established, its significance as a nutritional parameter is still a matter of debate. Furthermore, although albumin is known to be an acute-phase protein, there is a lack of evidence on how albumin levels change in the context of increasing inflammation and postoperative complications. Enhanced knowledge of whether and when albumin can be used as a nutritional parameter can improve nutritional assessment and may have consequences for decisions regarding enteral or parenteral nutritional therapy and on the timing of elective surgery.

The aim of this study was twofold. First, we aimed to investigate whether albumin is a useful nutritional marker in patients scheduled for elective intestinal surgery. Second, we intended to further investigate albumin levels in the postoperative phase with regard to major complications and inflammation.

## 2. Methods

### 2.1. Ethical Approval

Ethical board approval was obtained from the Medical Ethics Commission II of the Medical Faculty Mannheim, Heidelberg University, Mannheim, Germany (2017-554N-MA). Written informed consent was obtained from all patients included in this study. All investigations were performed according to the Declaration of Helsinki.

### 2.2. Study Population

Between April 2017 and August 2018, elective patients undergoing intestinal surgery at our institution were offered a preoperative nutritional risk screening (NRS 2002) and measurements of handgrip strength (HGS), body mass index (BMI), and phase angle (PA) in addition to the routine measurements of serum albumin and C-reactive protein (CRP). Exclusion criteria were patients who were not willing or able to provide written informed consent, patients under 18 years of age, and patients undergoing nonelective procedures. All patients who took part in these measurements were eligible for this study.

### 2.3. Patient Characteristics

Further patient characteristics and data on surgery were collected from medical records. Concerning NRS, patients were regarded as nutritionally at risk when a score ≥3 was obtained [17]. Preoperative blood samples for the determination of serum albumin and CRP were routinely acquired on the day of admission. In the postoperative course, blood samples were routinely collected on postoperative day (POD) 1/2 and on POD 4/5, depending on the clinical need of the patient. Postoperative complications were recorded according to the Clavien–Dindo classification [18]. Major complications were defined as those with a Clavien–Dindo grade ≥ III. For evaluation of the relative change of serum albumin (∆-albumin) in patients with a postoperative decrease in albumin levels, the following definition was used: (albumin level before surgery−albumin level after surgery)/albumin level before surgery × 100%.

### 2.4. Handgrip Strength

Handgrip strength (HGS) was determined using a Saehan Hydraulic Hand Dynamometer SH5001 (Saehan Corporation, Changwon, South Korea). For measurements, patients sat in a comfortable position with a 90° angle at the elbow joint and were asked to squeeze the dynamometer using their maximum strength, and the test was repeated after a break of 30 seconds. In total, three measurements of the dominant hand were obtained and averaged.

### 2.5. Phase Angle

Bioelectrical impedance analysis (BIA) measurements were conducted to obtain PA values. In brief, after physical rest for 30 minutes, four gel electrodes were applied, with two detecting electrodes placed at the ulnar aspect of the wrist and the medial malleolus of the dominant body side of the patients. Measurements were conducted and recorded by a multiple frequency BIA instrument following standard protocols (Nutriguard-M, Data Input GmbH, Frankfurt, Germany). Body composition values were calculated using Nutriguard Plus software (version 5.4, Data Input GmbH).

### 2.6. Skeletal Muscle Mass Index

The skeletal muscle mass index (SMI) was derived from the muscle mass determined by computed tomography (CT) or magnetic resonance imaging (MRI) studies conducted for routine diagnostic purposes up to 8 weeks before surgery. Two adjacent axial images within the same series were selected, and the total muscle cross-sectional area (cm^2^) at the third lumbar vertebra (L3) was determined and averaged for each patient. The following muscles were selected using aycan workstation pro software (version 3.12.000, aycan Digitalsysteme GmbH, Würzburg, Germany): rectus abdominis, abdominal (lateral and oblique), psoas, and paraspinal muscles (quadratus lumborum and erector spinae). The muscle area in centimeters squared (cm^2^) was calculated and then normalized by patient height in meters squared (m^2^) and reported as lumbar SMI (cm^2^/m^2^).

### 2.7. Statistical Analysis

Mean and standard deviation (SD) were calculated for each quantitative variable. Qualitative variables are expressed as absolute and relative frequencies. Student's *t-*test was used to compare the mean values of approximately normally distributed quantitative variables. The Mann–Whitney *U* test was used for quantitative variables that were not normally distributed. For qualitative variables, a *χ*^2^ test or Fisher's exact test was used, as appropriate. All statistical tests for the comparison of two groups have been performed as two-tailed tests. In general, a test result was considered statistically significant when *p* < 0.05. A multiple linear regression analysis was performed in order to analyze several factors associated with preoperative albumin levels simultaneously. Prior to multiple analyses, univariable comparisons were performed to investigate whether each factor was associated with the outcome. For correlation analyses, Pearson's correlation (r), Spearman's correlation (*r*_s_), or the point biserial correlation (r_pb_) were used, as appropriate. Furthermore, a receiver-operator characteristic (ROC) analysis was performed to determine optimal cut-off values for parameters separating patients with major postoperative complications from patients without major postoperative complications. Statistical analyses were performed using IBM SPSS Statistics (version 25, IBM Corp., Armonk, NY, USA) and GraphPad Prism version 8.1.0 (GraphPad Software Inc., San Diego, CA, USA).

## 3. Results

### 3.1. Patients' Characteristics

Between April 2017 and August 2018, 111 patients scheduled for intestinal surgery gave informed consent to preoperative measurements of nutritional parameters. In three patients, preoperative measurements were not possible due to technical reasons, and in another three patients, no intestinal procedure was performed. The data of the remaining 105 individuals were used in the analysis. The patients' characteristics are summarized in Table 1. Patients were equally distributed regarding sex and age with a mean age of 53.2 ± 16.1 years. The main indications for surgery were colorectal cancer (CRC) (37.1%), IBD (39.1%), and diverticular disease (7.6%). The operations performed included colectomies, hemicolectomies, and rectal resections as well as formations and reversals of ostomies. The majority of patients were classified as American Society of Anesthesiologists (ASA) grade 1 or 2 (76.0%). The majority of procedures were performed laparoscopically (68.8%).

### 3.2. Correlations of Albumin with Nutritional Status and Inflammation

**Figure 1:**
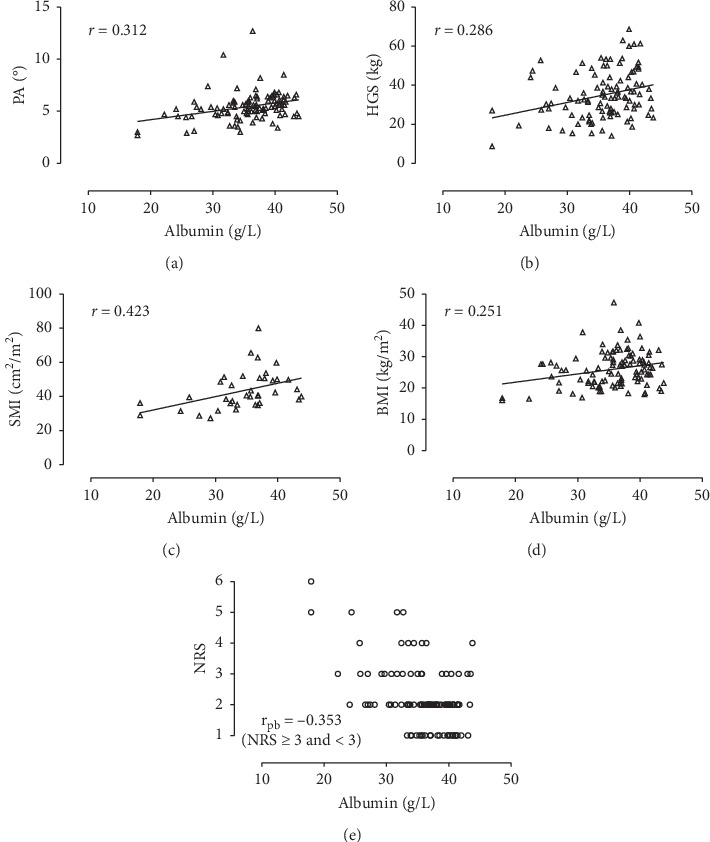
Preoperative setting, (a–d) dispersion graphs with linear regression lines depicting correlations of albumin with nutritional parameters and (e) scatter plot for the distribution of albumin values over NRS grades.

**Figure 2:**
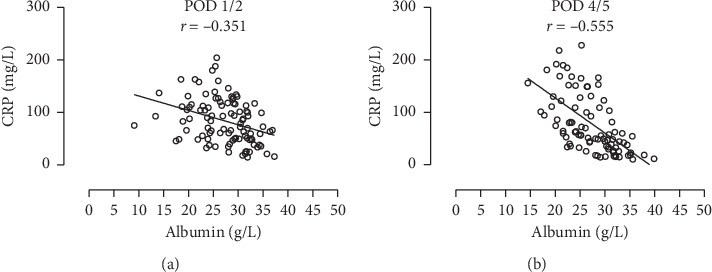
Scatter plots with linear regression lines depicting the increasing negative correlation between albumin and CRP values during the postoperative course (a) POD 1/2 and (b) POD 4/5 (one data point is outside the axis limits).

Preoperative measurements are listed in Table 2. Preoperative albumin levels were negatively correlated with CRP (*r*_s_ = −0.563; *p* < 0.001) and NRS scores ≥3 (*r*_pb_ = −0.353; *p* < 0.001) and were positively correlated with PA (*r* = 0.312; *p*=0.001), HGS (*r* = 0.286; *p*=0.003), BMI (*r* = 0.251; *p*=0.010), and SMI (*r* = 0.423; *p*=0.008). Dispersion graphs of correlations between preoperative albumin levels and nutritional parameters are shown in Figure 1. A multiple linear regression model was used to investigate the association of preoperative albumin with CRP, PA, HGS, BMI, and NRS simultaneously (Table 3). Only CRP (*p* < 0.001) and PA (*p*=0.004) were the independent factors influencing albumin levels (*R*^2^ = 0.472, *F*(2,99) = 44.177, and *p* < 0.001). In addition, a subgroup analysis of the patients for whom SMI was available was performed (*n* = 36). For these patients, CRP (*p* < 0.001) and PA (*p*=0.004) remained the two independent parameters predicting albumin values (*R*^2^ = 0.534, *F*(2,34) = 19.510, and *p* < 0.001). Postoperatively, an increase in correlation strength between albumin and CRP was observed from POD 1/2 (*r* = −0.351) to POD 4/5 (*r* = −0.555) (Figure 2).

### 3.3. Associations with Postoperative Outcomes

**Figure 3:**
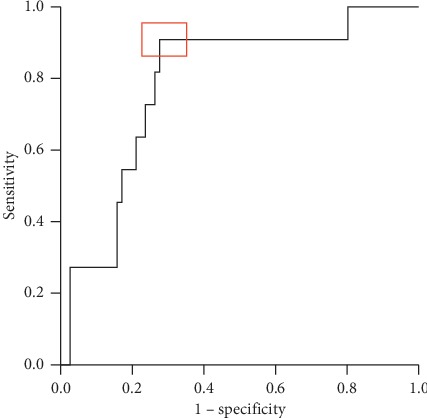
ROC curve for the determination of an optimal cut-off value for the product of ∆-albumin and CRP on POD 4/5 to identify patients with major complications (sensitivity 91% and specificity 72%; red marker).

Postoperative complications and the length of hospital stay are reported in Table 4. The overall rate of major postoperative morbidity was 12.4%. The mean hospital stay was 9 ± 4.5 days. The length of hospital stay was significantly longer (*p* < 0.001) for patients with major postoperative complications (16 ± 8.7 days) compared with patients without major postoperative complications (8 ± 2.5 days). Neither preoperative albumin (*p*=0.843) nor preoperative CRP (*p*=0.342) showed a statistically significant association with the occurrence of major postoperative complications. However, the ∆-albumin from the preoperative to the first (∆_Alb1_, *p*=0.025) and second postoperative measurement (∆_Alb2_, *p*=0.003) was significantly greater in patients with major postoperative complications as compared with patients without major postoperative complications. A ROC analysis was performed and a cut-off value for ∆_Alb1_ of 27.3% was calculated to separate patients with high-grade morbidity from patients without high-grade morbidity (sensitivity 75%, specificity 69%, and Youden's index 0.44). For ∆_Alb2_, this cut-off value was 24.3% (sensitivity 91%, specificity 55%, and Youden's index 0.46). At POD 4/5, the CRP values of patients with major complications were significantly higher as compared with patients without major complications (*p*=0.003) with a cut-off value of 67.3 mg/L (sensitivity 92%, specificity 63%, and Youden's index 0.55). When the product of ∆_Alb2_ and CRP on POD 4/5 was calculated, a more reliable identification of patients with high-grade morbidity than with ∆_Alb2_ or CRP alone was achieved (sensitivity 91%, specificity 72%, and Youden's index 0.63). The corresponding ROC curve is shown in Figure 3.

## 4. Discussion

In this study, we investigated albumin, nutritional parameters, and inflammation in the perioperative course of 105 patients undergoing elective intestinal surgery between April 2017 and August 2018. Preoperatively, serum albumin was a marker of nutritional status even if an inflammatory component was present. However, when inflammation increased during the early postoperative course, albumin levels were increasingly correlated with CRP, thus no longer suitable as a nutritional marker. A significant association with surgical outcomes was found for the reduction of albumin levels from the preoperative day to POD 1/2 and POD 4/5, and a new surrogate marker for postoperative morbidity was calculated.

The correlation of preoperative albumin levels with several different established nutritional biomarkers (PA, HGS, SMI, BMI, and NRS) indicated that preoperative albumin was a marker of nutritional status in patients scheduled for elective intestinal surgery. This is an important finding as the suitability of albumin as a nutritional parameter was questioned in several previous publications [12–15]. Moreover, this has further implications for the perioperative management of surgical patients as low preoperative albumin levels may identify not only patients at risk for high-grade morbidity but also those who might benefit from nutritional support, thus justifying the postponement of elective surgical procedures in favor of nutrition therapy.

Concerning nutritional status, a gold standard for its evaluation does not exist [19]. We measured PA, HGS, BMI, and SMI and conducted a NRS 2002 nutritional risk screening to assess nutritional status because evidence for aptitude of these parameters as nutritional markers is available [17, 20–23]. To measure inflammation, we used CRP, which has been proven as a robust marker of inflammatory processes [24]. Preoperatively, albumin was correlated with both nutritional parameters and CRP. In multivariable analysis, PA, which is regarded as one of the most reliable parameters to assess nutritional status, and CRP were the only independent factors influencing albumin levels prior to surgery. From this, we conclude that albumin may be regarded as a nutritional parameter in the preoperative course of patients undergoing elective intestinal surgery, even if a certain inflammatory component is present.

During the acute phase of the postoperative course, we observed an increase in the strength of the negative correlation of albumin with CRP, reflecting the role of albumin as an acute-phase protein [25]. In the view of increasing inflammation, the significance of albumin as a nutritional parameter is biased; it should therefore not be used to assess nutritional status under these clinical conditions. As a hepatic serum protein, albumin is affected not only by nutritional state and inflammation but also by liver function and volume status [26]. Here, we focused on the influence of nutrition and inflammation on albumin levels. Concerning possible confounders, we investigated a cohort undergoing elective, nonhepatic procedures with the majority of patients graded ASA 1 or 2 (76.0%) with a mean preoperative bilirubin value of 0.54 ± 0.54 mg/dL and a mean preoperative creatinine value of 0.93 ± 0.29 mg/dL. Furthermore, preoperative administration of intravenous fluids is not part of the standard operation procedure for patients undergoing elective intestinal resections at our institution. Therefore, impaired liver and renal functions, as well as hypervolemia, are rather theoretical confounders of the preoperative albumin levels in this cohort.

We report an overall rate of Clavien–Dindo complications ≥ grade III of 12.4%. Most probably, because of the small number of major complications, we did not find a significant association of preoperative albumin levels with postoperative major complications, which has been reported in a number of larger studies [1, 2]. However, even in this setting where no significant association of preoperative hypoalbuminemia with major postoperative morbidity was observed, ∆-albumin values were significantly associated with postoperative major complications. Of note, this applied also to ∆-albumin on POD 1/2, thus allowing early identification of patients with a high probability to develop postoperative major complications. These findings emphasize the importance of serum albumin as a surrogate parameter for major postoperative morbidity and are in line with results of a recent retrospective analysis of 626 patients undergoing colorectal resection [27]. The product of ∆-albumin and CRP on POD 4/5 identified patients with major complications with high sensitivity and specificity and performed better than ∆-albumin or CRP on POD 4/5 alone. This is in accordance with clinical tools such as the Glasgow Prognostic Score, a prognostic indicator for various types of cancers calculated with CRP and albumin values [28–30].

Some limitations of our study must be mentioned. Although preoperative data on nutritional parameters were prospectively collected, the study was retrospective in nature and not all consecutive patients during the study period could be included. Perioperative treatment was not controlled but followed standard operating procedures. The cohort was inhomogeneous regarding diagnoses and surgical procedures. However, the patients in this study represent the spectrum of elective intestinal surgery at a tertiary referral center, with the majority of patients operated for CRC, IBD, and diverticular disease. Therefore, we investigated albumin levels in a cohort in which its prognostic value has been demonstrated in previous studies [1, 2, 5, 6].

## 5. Conclusion

First, our study demonstrates that albumin may be regarded as a surrogate marker for nutritional status prior to elective intestinal surgery despite contradictory reports in the past. This also seems to be true when a certain chronic inflammatory component is present, which is common in surgical patients diagnosed with CRC or IBD. We also show that, in the acute postoperative phase, albumin should not be used as a nutritional parameter due to an increasing correlation with inflammation. Therefore, assessment of the patients' inflammatory state is essential before using albumin as a nutritional marker. Second, even in a cohort where preoperative albumin did not predict postoperative complications, ∆-albumin identified patients at risk for major morbidity, underlining the importance of albumin as a prognostic tool. The product of ∆-albumin and CRP on POD 4/5 could be a useful parameter to reliably identify patients with high-grade morbidity.

## Figures and Tables

**Table 1:** Patients' characteristics.

Category	Characteristic	n or mean	% or SD
General	Female	50	47.6
	Male	55	52.4
	Age	53.2	±16.1

Indication	IBD	41	39.1
	CRC	39	37.1
	Other malignancies	3	2.9
	Diverticular disease	8	7.6
	Other	14	13.3

Surgery	Ileocecal resection	9	8.6
	Right hemicolectomy	8	7.6
	Left hemicolectomy/sigmoid resection	12	11.4
	Rectal resection	10	9.5
	Abdominoperineal resection	3	2.9
	Proctocolectomy/subtotal colectomy	18	17.1
	Reversal of loop ileostomies	22	21.0
	Reversal of other ostomies	10	9.5
	Other	13	12.4

Surgical approach^a^	Laparoscopy^b^	55	68.8
	Conversion^c^	9	11.3
	Primarily open	16	20.0

Type of anastomosis	Small bowel–small bowel	26	24.8
	Small bowel–large bowel	33	31.4
	Large bowel–large bowel	32	30.5
	None	14	13.3

^a^stoma reversal via ostomy excluded (no laparoscopy and no laparotomy); *n* = 80. ^b^laparoscopic or laparoscopically assisted. ^c^of laparoscopically intended procedures. IBD, inflammatory bowel disease; CRC, colorectal cancer.

**Table 2:** Preoperative measurements.

Category	Characteristic	n or mean	% or SD
Biological data	BMI (kg/m^2^)	26.1	±5.6
	PA (°)	5.4	±1.4
	HGS (kg)	35.0	±12.3
	SMI (cm^2^/m^2^)	43.5	±11.0
	Albumin (g/L)	35.7	±5.4
	CRP (mg/L)	13.0	±20.6
	Bilirubin (mg/dL)	0.54	±0.54
	Creatinine (mg/dL)	0.93	±0.29

NRS	1	23	21.9
	2	51	48.6
	3	19	18.1
	4	7	6.7
	5	4	3.8
	6	1	1.0

ASA	1	8	7.7
	2	71	68.3
	3	23	22.1
	4	2	1.9

BMI, body mass index; PA, phase angle; HGS, handgrip strength; NRS, nutritional risk score.

**Table 3:** Factors predicting preoperative albumin level in multivariable analysis.

Analysis	Parameter	*β*	*p* value
*Entire cohort*			
Factors in the model	CRP	−0.619	<0.001^*∗*^
	PA	0.218	0.004^*∗*^

Factors not in the model	HGS	0.097	0.206
	NRS	−0.027	0.752
	BMI	0.124	0.095

*SMI subgroup*			
Factors in the model	CRP	−0.511	<0.001^*∗*^
	PA	0.385	0.004^*∗*^

Factors not in the model	HGS	0.075	0.569
	NRS	−0.162	0.225
	BMI	0.129	0.284
	SMI	0.205	0.099

PA, phase angle; HGS, handgrip strength; NRS, nutritional risk score; BMI, body mass index; SMI, skeletal muscle mass index.

**Table 4:** Postoperative outcomes.

Category	Characteristic	n or mean	% or SD
Clavien–Dindo grade	I	21	20.0
	II	12	11.4
	IIIa	4	3.8
	IIIb	6	5.7
	IVa	2	1.9
	IVb	0	0
	V	1	1.0

Major complications	No	92	87.6
	Yes	13	12.4

Hospital stay	Days	9	±4.5

## Data Availability

The patient data used to support the findings of this study are restricted by the Medical Ethics Commission II of the Medical Faculty Mannheim, Heidelberg University, Mannheim, Germany, in order to protect patient privacy. Data are available from the corresponding author for researchers who meet the criteria for access to confidential data.
